# Supplementary material for: Stepwise remodeling and subcompartment formation in individual vesicles by three ESCRT-III proteins
Source: iScience. 2022 Dec 8;26(1):105765. doi: 10.1016/j.isci.2022.105765 (PMC9800321; doi:10.1016/j.isci.2022.105765)
Supplement: Document S1. Figures S1–S11 and Table S1 [file mmc1.pdf]

iScience, Volume *26*

Supplemental information

Stepwise remodeling and subcompartment formation in individual vesicles by three ESCRT-III proteins

Yunuen Avalos-Padilla, Vasil N. Georgiev, Eleanor Ewins, Tom Robinson, Esther Orozco, Reinhard Lipowsky, and Rumiana Dimova

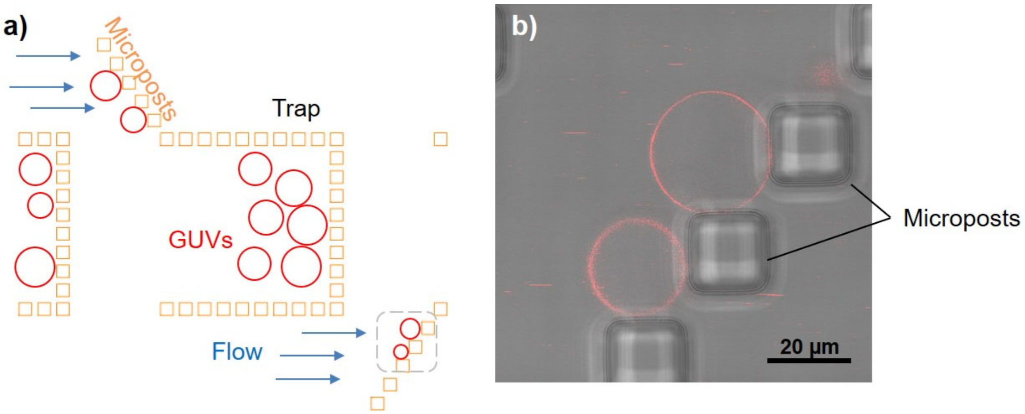

Figure S1. Vesicle trapping in a microfluidic device, related to Figure 1. (a) A sketch of part of the microfluidic chip reported previously [S1], showing a trap and the microposts by which individual vesicles can be stopped and monitored. (b) An overlay of confocal and phase-contrast image showing two vesicles trapped by the posts in a microfluidic device (as illustrated by the dashed-line region indicated in panel a). The flow direction of introduced solution is from the left.

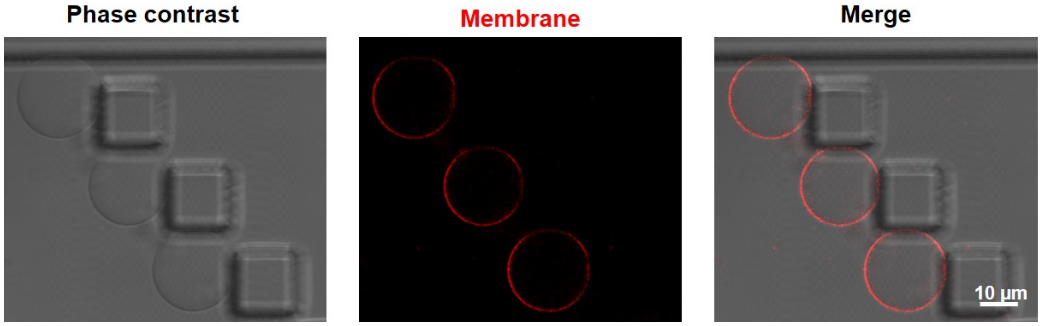

Figure S2. Buffer flushing and incubation of GUVs does not result in ILV formation, related to Figure 1. POPC:POPS:Chol:PI(3)P (62:10:25:3) GUVs were incubated with equivalent protein-free buffer (25 mM Tris, 150 mM NaCl, pH = 7.4) volumes as in Figure 1. Images show 3 GUVs after six rounds of buffer exchange, similar to the conditions shown in Figure 1.

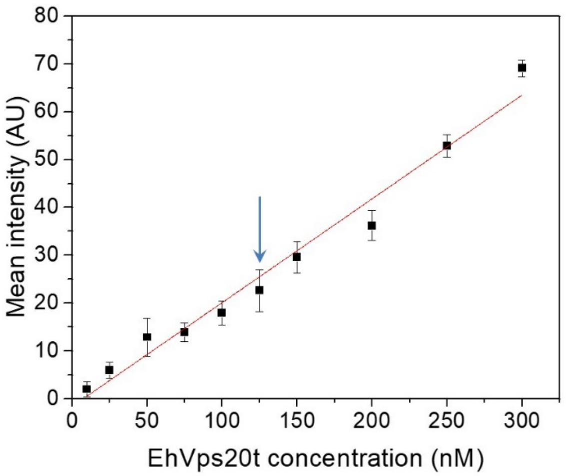

Figure S3. Linear dependence of the bulk fluorescence intensity of OG-EhVps20t, related to Figure 2. OG-EhVps20t mixed with unlabelled EhVps20t (1:4, v:v) dissolved in 25 mM Tris, 150 mM NaCl, pH = 7.4 at room temperature. Arrow points to the typical working concentration. The red line is a linear fit. The error bars represent the standard deviations of 20 GUVs observed for each condition.

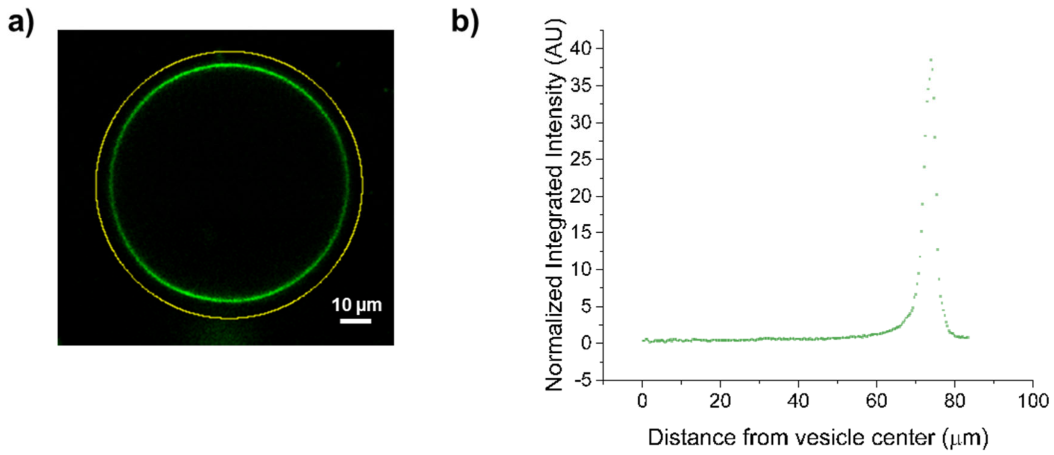

Figure S4. Standard curve generation, related to Figure 2. (a) Representative confocal image of a vesicle labelled with OG-DHPE used for the standard curve generation; the polarization effect is visible from the angular dependence of the florescence. (b) Typical intensity radial profile. The value of intensity was obtained by measurement of the area under the curve generated in the plot.

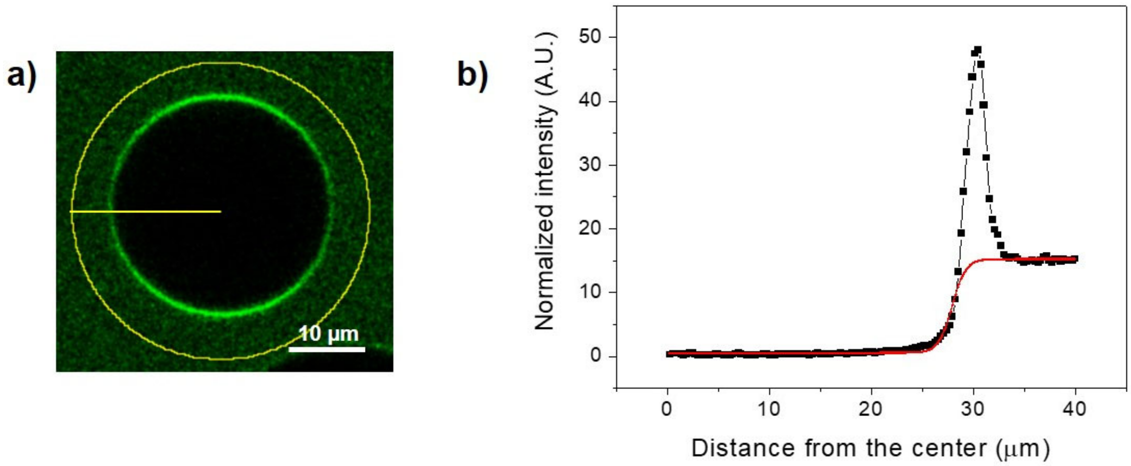

Figure S5. OG-EhVps20t coverage measurement, related to Figure 2. (a) Typical confocal image of a GUV used for measuring the coverage concentration of OG-EhVps20t at the membrane. (b) Radial intensity profile (black squares) and subtracted intensity of the non-adsorbed protein in the bulk (red curve).

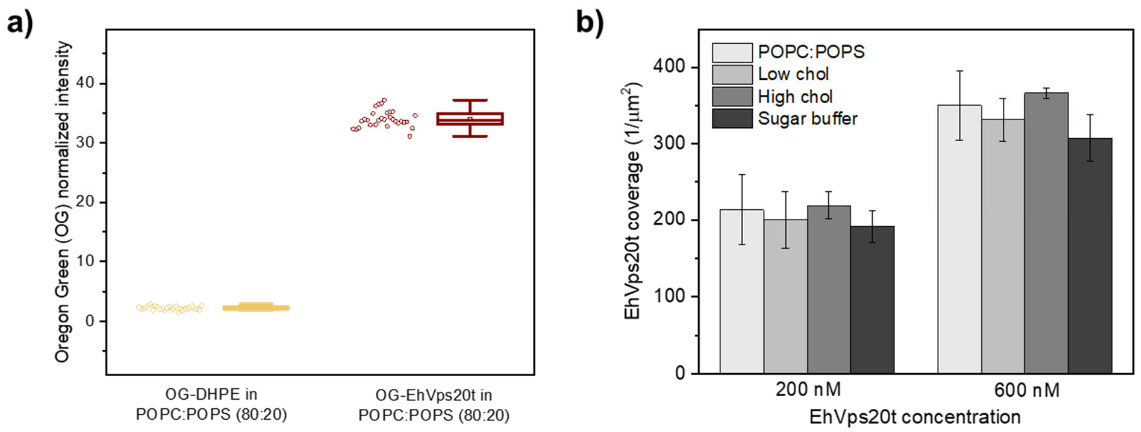

Figure S6. Assessing the EhVps20t coverage from intensity measurements, related to Figure 2. (a) To account for the different quantum yield of OG in the labeled lipid and the protein, the intensity of lipid solutions and that of the protein in the presence of the lipid solutions at identical settings of the microscope were evaluated (see STAR Methods). Each symbol (on the left side of the boxes) corresponds to an individual measurement. The samples were diluted in the normal protein buffer (25 mM Tris, 150 mM NaCl, pH = 7.4). The signal is normalized by the concentration of OG. (b) Protein coverage of EhVps20t at two different concentrations of the protein present in the bulk. In order from left to right, different types of conditions were tested (1) POPC:POPS (80:20) GUVs, (2) POPC:POPS:Chol:PI(3)P (62:10:25:3) GUVs (Low chol) and (3) POPC:POPS:Chol:PI(3)P (6:10:35:3) GUVs (High chol), incubated in the normal protein buffer (25 mM Tris, 150 mM NaCl, pH = 7.4). The darker bar, represent results from POPC:POPS:Chol:PI(3)P (62:10:25:3) GUVs incubated in sugar buffer (600 mM Sucrose). At least twenty vesicles were measured for each condition, standard errors are shown for each bar.

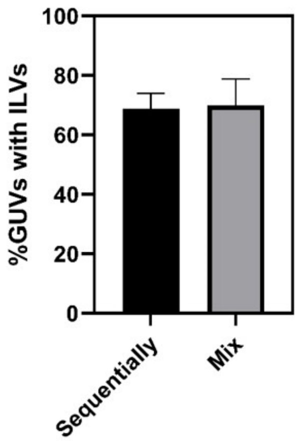

Figure S7. Effect on ILV formation by protein addition approach – sequentially or premixed, related to Figure 3**.** POPC:POPS:Chol:PI(3)P (62:10:25:3) GUVs were incubated with 125 nM of EhVps20t, 600 nM of EhVps32 and 300 nM EhVps24, either sequentially leaving 5 min of incubation between each protein addition or together in a mix leaving 15 min incubation at the end. Data represent the mean and standard error of three independent experiments where 30 GUVs of each replicate were screened.

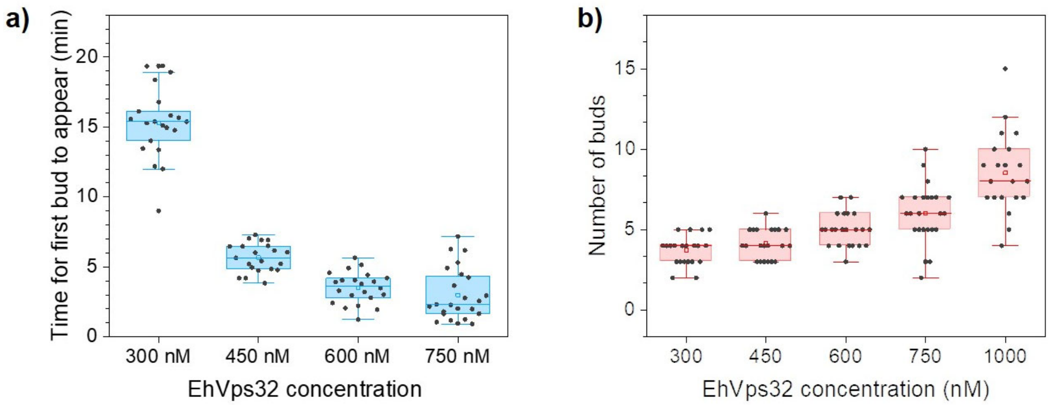

Figure S8. Kinetics of bud formation regulated by EhVps32 concentration, related to Figure 4. POPC:POPS:Chol:PI(3)P (62:10:25:3) GUVs were incubated with 125 nM of EhVps20t for 5 min followed by different concentrations of EhVps32 as shown in the x-axis. At least 10 vesicles of three independent preparations were followed over time in a microfluidic device and (a) the time that took for the first bud to be observed is plotted. At higher concentrations of EhVps32, namely 1000 nM, the process was faster than 1 minute and the exact time frames could not be determined because of difficulties in experimental handling. (b) Total number of buds detected in each GUV after 30 min of incubation with EhVps32. Black data points show the single measurements for each condition. Boxes show the range from 25-75% of data and the median line.

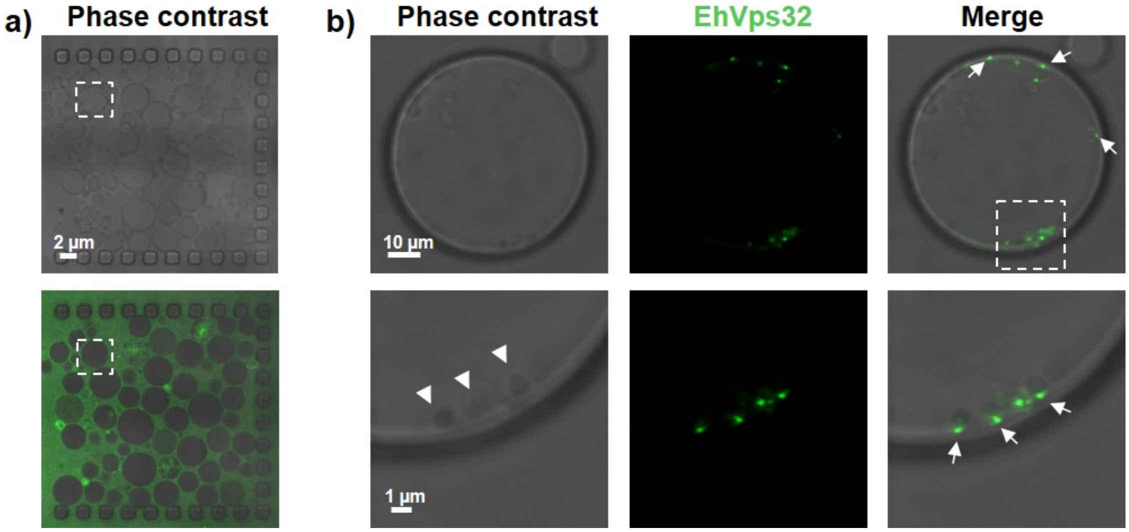

Figure S9. EhVps32–enriched domain formation in GUVs, related to Figure 4. (a) POPC:POPS:Chol:PI(3)P (62:10:25:3) GUVs were loaded on a microfluidic chamber at a flow rate of 10 µl/min. Then 125 nM of EhVps20t and 300 nM of EhVps32 (20% of EhVps32 was labelled) were subsequently flushed at a constant rate of 0.1 µl/min. (b) Close-up views of the vesicle in the dashed region in (a) after the excess of proteins was washed away, showing protein-domain formation (arrows) in the site where buds (arrowheads in phase-contrast image) are generated.

	EhVps32 (nM)		
**EhVps20t (nM)**	**300**	**600**	**1000**
**125**	2.46 ± 0.64[a]	1.29 ± 0.28[b]	1.05 ± 0.25[c]
**300**	3.51 ± 0.56[d]	3.08 ± 0.49[e]	2.35 ± 0.40[a]
**600**	4.11 ± 0.31[f]	3.70 ± 0.64[d]	2.53 ± 0.44[a]

Table S1. Statistical analysis of the data in Figure 5 in the main text. Data are means ± SE of ILVs diameter measured in at least 20 GUVs. Significant differences ($p < 0.05$, Tukey's test) calculated using one-way ANOVA are denoted by different letters: shared letters represent no statistically significant difference as evaluated with the SPSS statistics software for Windows v17.0 (Chicago: SPSS Inc.).

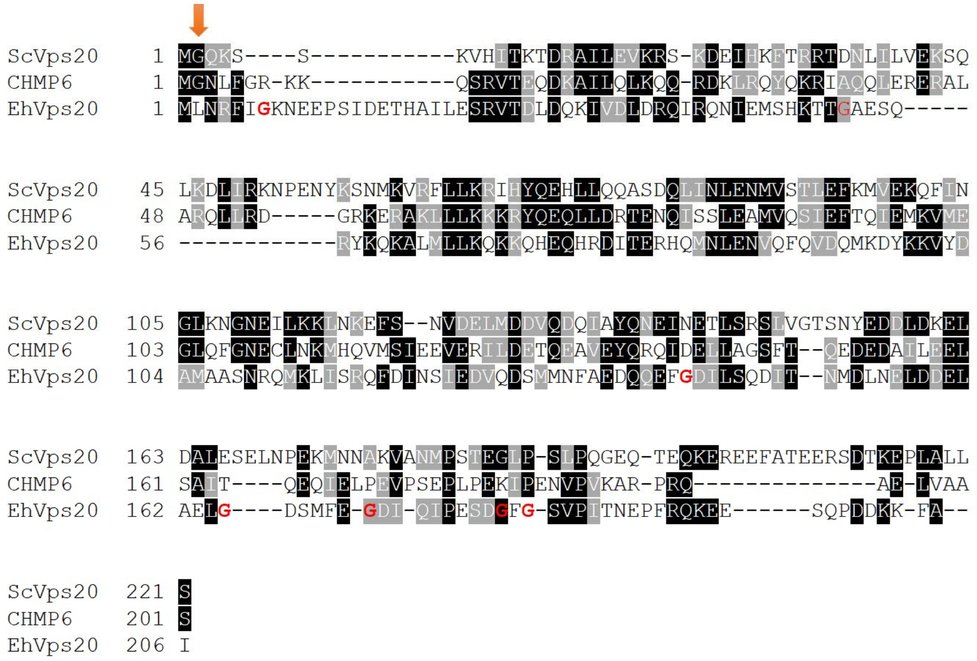

Figure S10. Blast analysis of EhVps20 with its human and yeast homologues, related to Figure 5. EhVps20 lacks the glycine residue in its N-terminal necessary for protein myristoylation. Multiple sequence alignment for Vps20-homologues in yeast (ScVps20), human (CHMP6) and *E. histolytica* (EhVps20). Glycine residues present in EhVps20 are displayed in red. Arrow indicates the N-terminal glycine necessary for protein myristoylation. Sequence alignments were performed with Clustal Omega and edited in the BoxShade server (https://embnet.vital-it.ch/software/BOX_form.html).

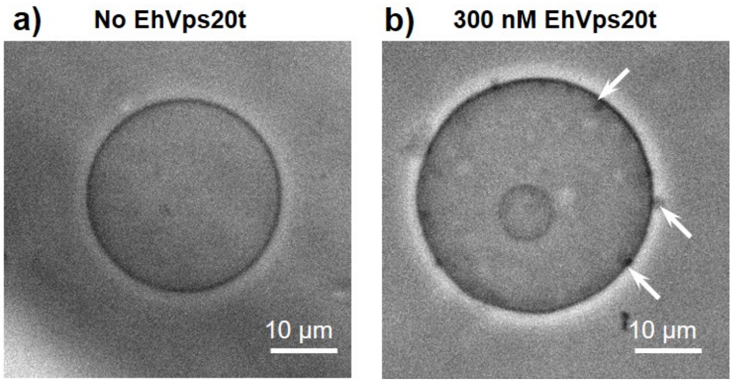

Figure S11. EhVps20t clusters in the membrane of GUVs at high protein concentrations, related to Figure 5. Electroformed POPC:POPS:Chol:PI(3)P (62:10:25:3) GUVs incubated in (a) a isoosmolar sucrose buffer (no EhVps20t) or (b) 300 nM of EhVps20t, observed under phase contrast. Arrows show protein clusters at the membrane.

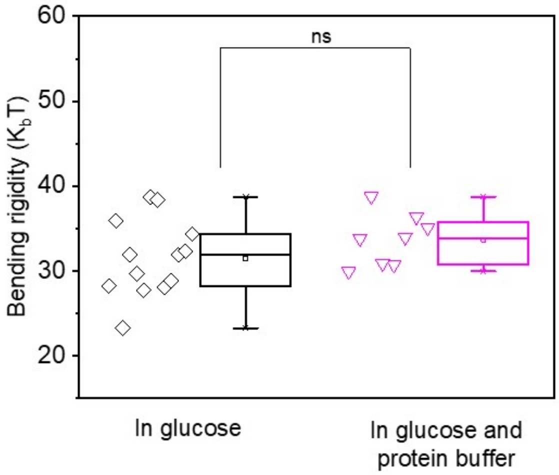

Figure S12. Bending rigidity of protein-free membranes, related to Figure 5. The vesicles were formed of POPC:POPS:Chol:PI(3)P (62:10:25:3) in 20 mOsm/Kg sucrose solution. The GUVs for the data set indicated by the black diamonds (the same data as "Low Chol" in Figure 5c in the main text) were diluted in isotonic glucose solution, while the vesicles used for the data in magenta triangles were diluted in a solution containing protein buffer and glucose. The latter solution represents the scenario when 125 nm EhVps20t was used (see Figure 5c in the main text).

Reference

1. Yandrapalli, N., and Robinson, T. (2019). Ultra-high capacity microfluidic trapping of giant vesicles for high-throughput membrane studies. Lab Chip *19*, 626-633.
